# Application of electrospinning technique in development of intelligent food packaging: A short review of recent trends

**DOI:** 10.1002/fsn3.1781

**Published:** 2020-07-21

**Authors:** Masoud Aman Mohammadi, Seyede Marzieh Hosseini, Mohammad Yousefi

**Affiliations:** ^1^ Department of Food Science and Technology National Nutrition and Food Technology Research Institute Faculty of Nutrition Sciences Food Science and Technology Shahid Beheshti University of Medical Sciences Tehran Iran; ^2^ Student Research Committee Department of Food Science and Technology National Nutrition and Food Technology Research Institute Faculty of Nutrition Sciences Food Science and Technology Shahid Beheshti University of Medical Sciences Tehran Iran; ^3^ Department of Food Science and Technology Faculty of Nutrition and Food Science Tabriz University of Medical Sciences Tabriz Iran

**Keywords:** electrospinning, food, intelligent packaging, nanofiber

## Abstract

Intelligent food packaging refers to packages with the ability to sense foodstuff changes and to inform customers of the packaging content variations. They are often accompanied by smart detecting devices. Providing a suitable platform to include these devices into packaging polymers has always been discussing. Electrospun nanofibers produced through the electrospinning have been recently utilized as an outstanding and novel platforms for this purpose. Thus, the main aim of this study is to investigate recent trends in producing intelligent food packaging using electrospinning technique. In this regard, this paper was categorized into two chief sections, including (a) the principal of electrospinning technique to fabricate fine nanofibers and the parameters affecting the quality of electrospun fibers, and (b) the role of nanofibers as a platform to cover pH indicators in intelligent food packaging.

## 1 INTRODUCTION

Intelligent packaging as a newly emerging food protection has attracted scientists and consumer attentions. Such packaging is able of detecting, recording, tracking, communicating, and providing necessary information of food quality to ensure consumer safety. They are usually accompanied with smart devices. The most universally used smart devices in intelligent packaging include the following: (a) data carriers, (traceability during production to distribution with bar codes and Radio Frequency Identification (RFID) tags); (b) indicators (detecting food safety and quality); and (c) sensors (fast and definite measurement of the analytes in foods). The global active and intelligent packaging market is expected to grow at a Compound Annual Growth Rate (CAGR) of more than 4% during 2018–2024 (DUBLIN, 2019). The active and intelligent packaging market was valued at USD 17.5 billion in 2019 and projected to grow to USD 25.16 billion by 2025, at a CAGR of 6.78% during the forecast period of 2020–2025 (www.mordorintelligence.com).

**FIGURE 1:**
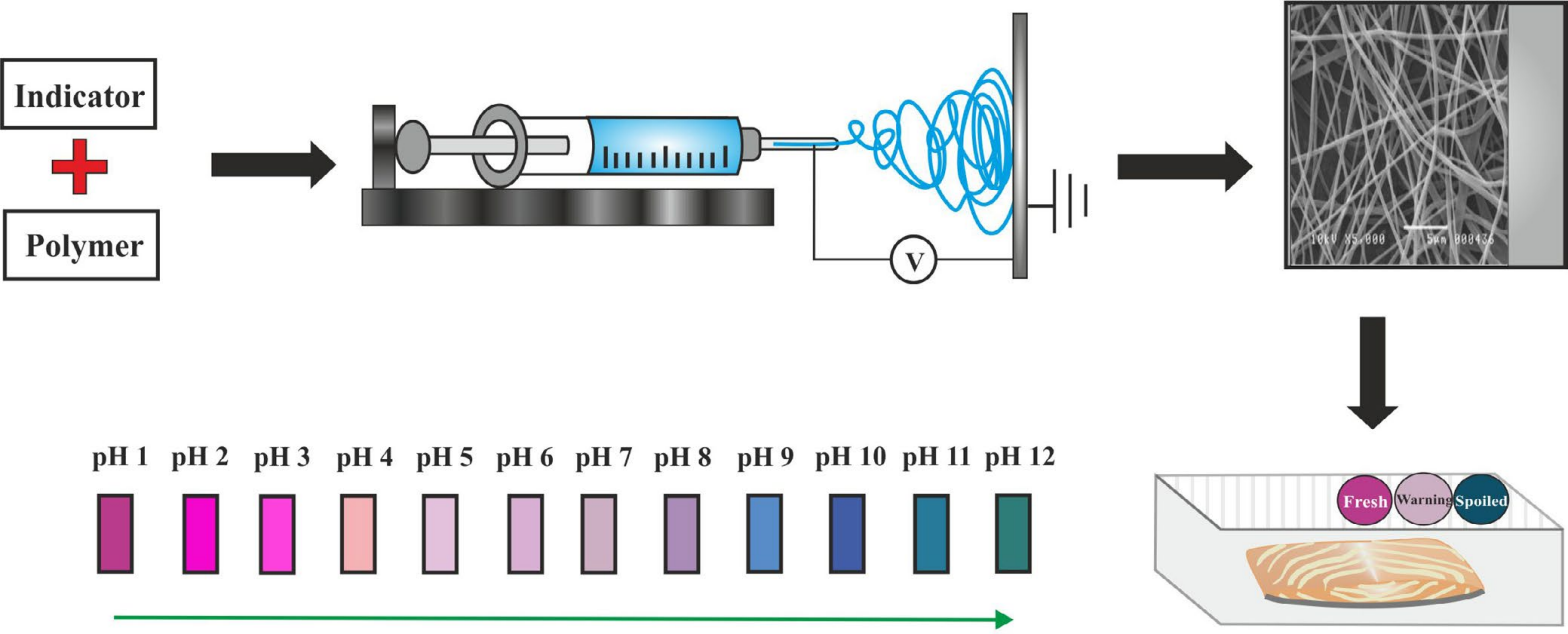
A schematic of application of electrospinning in intelligent food packaging

One of the newest methods for production of intelligent packaging is the electrospinning technique. Electrospinning is a simple, efficient, and low‐cost technique capable of fabricating nonwoven fibers, usually in submicron or nanoscale diameters (Mehta et al., 2017). In a contrast to other methods, this process could synthesize fibrous materials with higher surface areas and porosity, more responding to the surrounding environment variations, and high potential in controlling the release of incorporated compounds (Li et al., 2018). Therefore, electrospun nanofibers are considered as suitable platforms for covering indicators in intelligent packaging, though, their application in industrial scale is yet to come. A schematic of application of electrospinning in intelligent food packaging is given as Figure 1. In this review paper, we discuss recent uses of electrospinning technique in developing the intelligent packaging containing nanofibers covering pH indicators.

## 2 ELECTROSPINNING PRINCIPLES

Electrospinning has stemmed from the works for fabricating nanofibers used in filtering and textile applications in the early 19th century. Since then, it has seen remarkable progresses in terms of processing methods, factors, expanded materials, and uses. These developments have been brought about to the worldwide acceptance of electrospinning as a practicable method for fabricating nanofibers for different purposes (Liu et al., 2019; Niu et al., 2020; Norris et al., 2020; Sun, Perry, & Schiffman, 2019; Xue, Wu, Dai, & Xia, 2019). It is a simple, cost‐effective, flexible, unique, and suitable technique for large‐scale manufacturing of nanofibers (Homocianu & Pascariu, 2019). This technique is not only worthwhile in producing polymeric nanofibers, but also in the production of nanofibers from metals, ceramics, metal oxides, inorganic, and organic composite materials (Zhao, Lu, & Wang, 2018). This is feasible by changing the traits of the operating, solution, and ambient conditions of the procedure. It is likely to handle the diameter of nanofibers as well, playing an indispensable role in the functional characteristics of the substances utilized in several applications (Zhang, Li, Wang, & Zhang, 2020a). Furthermore, nanofibers can be achieved in diverse forms such as hollow, core‐shell, and porous nanofibers.

**FIGURE 2:**
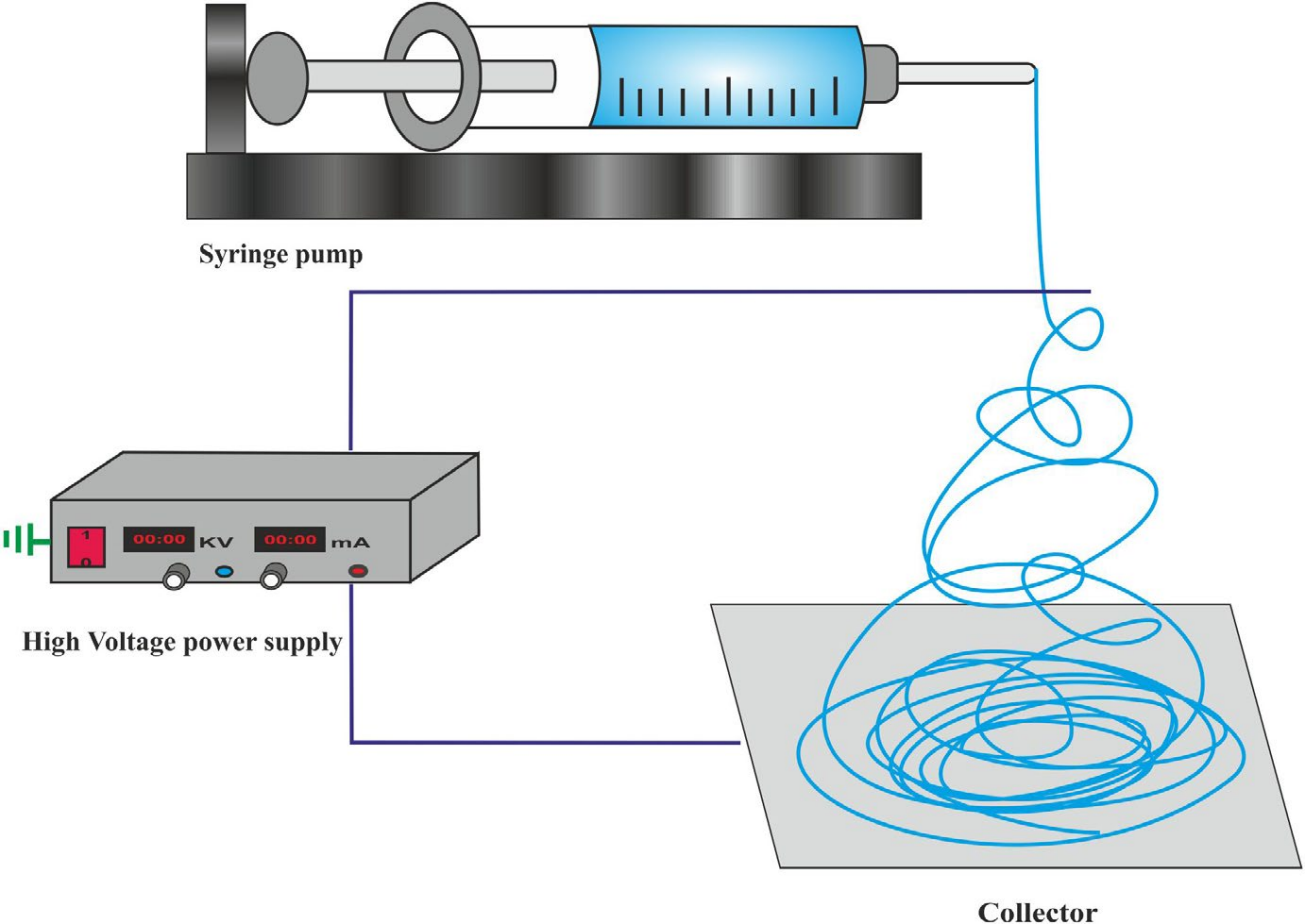
An illustration of an electrospinning device

An electrospinning device (Figure 2) is composed of a capillary tube or syringe, serving as the reservoir for keeping the polymer solution, a high voltage supplier, a metallic needle for dispensing the solution, and a collector to gather nanofibers (Janssen & Solberg, 2019). The needle and syringe are brought together and termed the needle assembly. The process takes place in 3 phases, called jet initiation, elongation, and solidification.

More detailed information regarding the function of electrospinning can be found in studies of Deshwal and Panjagari (2018), Rostami, Yousefi, Khezerlou, Aman Mohammadi, and Jafari (2019), Kotomin, Kulichikhin, and Skvortsov (2018), and Chiu et al. (2020).

## 3 PROPERTIES OF ELECTROSPUN NANOFIBERS

The properties of nanofibers are significantly affected by various processing parameters, such as the instrumental conditions (applied voltage, flow rate, and distance to the collector), polymer solution properties (viscosity, surface tension, conductivity, and solvent polarity), and the ambient conditions (temperature and humidity) (Maleki, Semnani Rahbar, Saadatmand, & Barani, 2018; Rostami et al., 2019).

### 3.1 Effect of instrumental conditions

**FIGURE 3:**
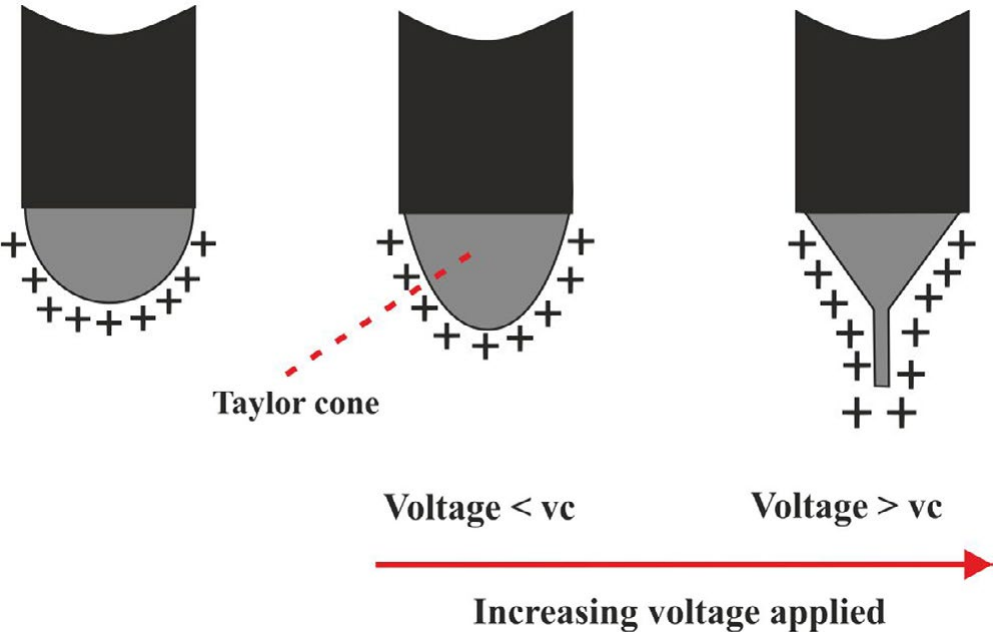
Figures of Taylor cone at different voltages (V_c_, Critical voltage)

The applied voltage plays an important role in the electrospinning process. At low voltages, nonuniform fibers with beads may be formed, but at high voltages, there is tends to slightly decrease the length of the single jet, increase the apex angle of the Taylor cone, and produce thicker and nonuniform fibers, and also greater amounts of charge in high voltage will lead to drawn faster and a greater volume of solution from the tip of the needle, resulting in a smaller and less stable Taylor cone (Figure 3) (Ghorani & Tucker, 2015; Haider, Haider, & Kang, 2018; Motamedi, Mirzadeh, Hajiesmaeilbaigi, Bagheri‐Khoulenjani, & Shokrgozar, 2017).

One of the other important parameters for successfully desirable electrospinning fibers is flow rate. The flow rate is the amount of polymer solution flowing from the needle to the Taylor cone per time (Xue et al., 2019). At low flow rates, the polymer solution will have adequate time for polarization, generating fibers with smaller diameters; while high flow rates result in thicker fibers with larger diameters owing to short drying time and low stretching force (Mercante, Scagion, Migliorini, Mattoso, & Correa, 2017). However, by the time the flow rate exceeds a critical level, the transferring rate of jet to the capillary tip surpasses the eliminating rate of the solution by the electrical force from the tip. This shift in the mass‐balance ends up in a sustained, but unstable jet, fabricating nanofibers with beaded structures (Chang, Chan, & Chang, 2016; Weng & Xie, 2015).

The distance between the tip and the collector (TCD) affects the diameter and morphology of the fibers also. Bead formation can be detected at a very long distance. Also, at a very short distance, the solvent cannot be completely evaporated before reaching the collector (Luo, Stoyanov, Stride, Pelan, & Edirisinghe, 2012), leading to production of wet fibers. The TCD should be optimized to allow sufficient time for the fibers to dry (Ghorani & Tucker, 2015). Long, Kamsom, Nurfaizey, Isa, and Masripan (2017) have shown that shorter tip‐collector distance produces wet, thick, and nonuniform fibers. In a little bite long distance, the fibers have more time for stretching and elongating before accumulating on the collector, leading to production of fibers with smaller diameter (Tong & Wang, 2007).

### 3.2 Effect of polymer solution properties

Molecular weight of polymer and its concentration are two parameters that determine the solution viscosity, which can influence the morphology of the fibers. Polymers with high molecular weights have usually the viscosity more than polymers with low molecular weights owing to the increased entanglements of polymer chains (Mercante et al., 2017). Decrease in the molecular weight or the concentration of polymer solution is associated with the production of fibers with surface defects (beads), but high concentrations or higher molecular weights lead to the greater solution viscosity and the formation of uniform fibers with a few beads. However, helix‐shaped, curly, and wavy fibers will be produced at very high concentration (Krumreich et al., 2019; Yang et al., 2004).

The surface tension influences nanofiber morphology as well. At low surface tension, beadless fibers can be formed. This factor depends on the polymer and solvent properties, being controlled by changing their mass ratio (Rogina, 2014; Xue et al., 2019). Uniform fibers can be produced when the surface tension of the solution is reduced at a fixed concentration (Fang, Yang, Yuan, Charlton, & Sun, 2017; Moreira, de Morais, de Morais, da Silva Vaz, & Costa, 2018).

The solution electrical conductivity is the other factor influencing the fibers morphology by facilitating the elongation of the droplets and the formation of a single or multiple jets (Thenmozhi, Dharmaraj, Kadirvelu, & Kim, 2017). Conductivity can be increased by addition of salts, ions, or conducting polymers to the polymer solution, which bring about the fabrication of high‐quality nanofibers with fewer defects and smaller diameters (Abutaleb et al., 2017; Afshari, 2016).

### 3.3 Effect of ambient conditions

The effect of the ambient conditions around the electrified jet has rarely been investigated. Humidity and temperature are thought to affect fiber morphology and the productivity of the electrospinning process (Haider et al., 2018). At high humidity, pores can be formed on the fiber surface (Casper, Stephens, Tassi, Chase, & Rabolt, 2004). Most of them studies report, that increasing the humidity, caused an increase in the number, diameter and distributed size pores (Cheng, Qin, Hu, Yu, & Zhu, 2017; Ramakrishnan et al., 2019; Shao, Yan, Chen, & Xiao, 2018).

Ambient temperature can significantly influence both the volatilization of the solvent and the viscosity of the polymer solution. Increasing the temperature provides a lower polymer solution viscosity and a higher solvent evaporation rate from the jet surface, which tends to produce of smaller diameter fibers. Shahabadi, Kheradmand, Montazeri, and Ziaee (2015) exhibited the effect of temperature on the morphology and diameter of electrospun fibers. They found that, as the temperature increased from 20 to 30°C, the diameter of the fibers decreased, and this reduction was related to the reduced viscosity of polymer solutions, increased solvent evaporation rate, and also high solubility of the polymer in the solvent.

## 4 APPLICATION OF ELECTROSPINNING IN FOOD PACKAGING

One of the essential processes used for maintaining the quality of foodstuffs in a period of production, transportation, and storage is packaging (Cruz, Alves, Khmelinskii, & Vieira, 2018). The main aim of packaging is preservation of food products from physical, chemical, and biological degradation (Ghoshal, 2018). Therefore, it delays the deterioration of foods as well as facilitating the transportation and distribution (Barska & WyrWA, 2017). The four basic functions that can be achieved by packaging are as follows: protection, convenience, containment, and communication (Ghaani, Cozzolino, Castelli, & Farris, 2016). For example, concerning its communication performance, packaging presents various information about food such as material type, color, shape, volume of the substance, and nutritional composition and calorie content to the consumer (Dalmoro et al., 2017).

Today, packaging technologies have been extensively developed, including active and intelligent packages to promote quality, safety, and the product shelf life (Fang, Zhao, Warner, & Johnson, 2017). One of the current technologies used in the food packaging is electrospinning. There are various techniques of forming nanofibers, such as high‐volume production procedures, including gas jet techniques, island‐in‐sea, and melt fibrillation, or highly cost methods, e.g. self‐assembly and nanolithography. Nevertheless, their usefulness is restricted by combinations of limited material ranges, feasible fiber assembly, production rate, and cost. Electrospinning has a privilege with its relatively high fabrication rate and comparative low cost (Ramakrishna et al., 2006).

Fundamental traits of electrospun fibers for detecting and sensing applications are including the 1D‐confinements features, the great orientation of structural components induced lengthwise the fibers, intensely constraint of both material and electronic diffusion perpendicular to nanofibers axis along with high porosities (up to 90%) (Mercante et al., 2017). The mixture of porosity and high surface area makes the opportunity to build multifunctional nanostructures capable of covering different indicators.

Despite these benefits, low throughput of electrospinning and the difficulty of processing in conventional equipment (due to high rigidity of biopolymers) and the low barrier property to moisture and oxygen (due to hydrophilicity biopolymers) has restricted industrial applications of electrospinning in packaging (Ding et al., 2019; Zhang, Li, Wang, & Zhang, 2020b). Also, because of the biodegradable nature of biopolymers used in food technologies, there is a lack of feasibility for a longer use or even a reuse of the electrospun biopolymer materials. The application of this technology in intelligent food packaging is discussed below.

### 4.1 Combination of electrospun nanofibers and indicators in intelligent food packaging

#### 4.1.1 Intelligent food packaging

According to The Commission of the European Communities, “intelligent food contact materials” are materials that monitor the condition of packaged food or the environment surrounding the food (Communities, 2004). Intelligent packaging is a system with one or more intelligent functions including monitoring, detecting, sensing, recording, tracing, and communicating during transport and storage, which promotes and enhances safety, quality, and shelf life of food products and also reports information about possible problems to the consumer or food manufacturers (Kalpana, Priyadarshini, Leena, Moses, & Anandharamakrishnan, 2019; Poyatos‐Racionero, Ros‐Lis, Vivancos, & Martínez‐Máñez, 2018). In order to achieve real‐time monitoring of a product during the supply chain, various smart devices, including indicators (for monitoring temperature, freshness, integrity, leakage, and pH), data carriers (bar codes) and sensors (gas sensors and biosensor) have been explored (Chowdhury & Morey, 2019; Ghaani et al., 2016; Müller & Schmid, 2019). This intelligent technology finds several applications in food spoilage detection, chemical contamination detection, anticounterfeit products, and pharmaceutical traceability (Bibi, Guillaume, Gontard, & Sorli, 2017; Dalmoro et al., 2017). Application of different smart devices in intelligent packaging of foodstuff is given in Table 1.

**TABLE 1:** Application of different smart devices in intelligent packaging of foodstuff

Type of smart device	Smart material	Feature	Packaging polymer	Food	References
Indicators	Alizarin	pH‐based	Chitosan	Fish	Ezati and Rhim (2020)
	*Echium amoenum*		Bacterial cellulose	Shrimp	Mohammadalinejhad, Almasi, and Moradi (2020)
	Black carrot		Cellulose‐chitosan	Pasteurized milk	Ebrahimi Tirtashi et al. (2019)
	Roselle		Starch Polyvinyl alcohol Chitosan	Pork	Zhang et al. (2019)
	Alizarin		Starch‐cellulose	Rainbow trout	Ezati, Tajik, Moradi, and Molaei (2019)
	Bromocresol green		Polypropylene	Rainbow trout	Rastiani et al. (2019)
	black carrot		Bacterial nanocellulose	Rainbow trout Common carp	Moradi, Tajik, Almasi, Forough, and Ezati, (2019)
	Black rice bran		Gelatin	Fish	Ge et al., (2019)
	Tamarind seed		Litmus lichen	Full cream milk	Liang and Wang (2018)
	Anthocyanin and poly‐lysine mixtures	CO_2_‐based	Nylon/LLDPE	Chicken breast	Saliu and Della Pergola (2018)
Sensor	Optical oxygen sensor	O_2_ determining	Polyethylene and ethylene‐vinyl acetate blend	Beef	Kelly, Santovito, Cruz‐Romero, Kerry, and Papkovsky (2020)
	Optical oxygen sensor (Optech‐O_2_ Platinum)	O_2_ determining	laminate plastic	Meat	Kelly, Cruz‐Romero, Kerry, and Papkovsky (2018)
	Radio Frequency Identification sensor	Dioxygen and carbon dioxide determining	Wheat gluten	Cheese	Saggin et al., (2019)

Biopolymers as well as plastics are widely used in intelligent food packaging. Even though various natural polymers have advantages (like biodegradable, renewable, and low cost) over plastics (Ghoshal, 2018; Khezerlou, Ehsani, Tabibiazar, & Moghaddas Kia, 2019), the full replacement of them with synthetic polymers seems not to be practical because of problems associated with technical and economical issues (Rogovina, Prut, & Berlin, 2019). Different biopolymers can be easily turned into a layer of nanofibers using the electrospinning method. This layer is utilized in two forms in food packaging, meaning as a single and independent layer (Akinalan Balik, Argin, Lagaron, & Torres‐Giner, 2019) or as an extra layer in the structure of biodegradable or synthetic plastic packages (Akinalan Balik et al., 2019; Pardo‐Figuerez, López‐Córdoba, Torres‐Giner, & Lagaron, 2018). In the both conditions, nanofiber layer can be employed as a platform for covering different compounds and substances such as food indicators. Anyway, going through the electrospinning‐related recent studies reveals the application of pH indicators as the only smart devices combined with electrospun nanofibers in intelligent food packaging. Electrospun fibers synthesized from polymer solutions are suitable candidates as platforms for coating different indicators by virtue of their high surface area, porosity, flexibility, absorption capacity, low‐cost production, and portable nature (Shen et al., 2011). Such fibers containing indicator materials show a high potential in food packaging owing to their high sensing sensitivity and fast response time, and thus enabling a better freshness evaluation of foods (Kalpana et al., 2019).

#### 4.1.2 Indicator

Indicators are defined as substances, determining the presence or the concentration of other substances or the measure of reaction between different substances by means of a specific change, particularly in color (Ghaani et al., 2016; Müller & Schmid, 2019). They can put across information to consumers that are related to the absence or presence of a specific substance, the level of a reaction between particles, or the concentration of a particular material. Indicators used in food packaging transport information to a consumer on the subject of food quality, microbial activity, or other characteristics of foods. In most cases, such information is exhibited by immediate visual variations, e.g., different intensities in color or the diffusion of dyes along the indicator geometry (Kalpana et al., 2019).

Indicators are although simple tools, but important in guaranteeing food safety, which allow a decrease in the loss of foodstuff and the cost of losses related to the repair or replacement of damaged products. In the broader senses, these smart devices could be categorized as internal and external indicators. The former represent indicators that are physically present inside the package and the latter represent indicators that are located outside the package (Pavelková, 2013). A distinct property of indicators is the kind of information they convey, which may be qualitative or semiquantitative. In spite of the large diversity of indicators, all of them can be categorized within three categories of freshness indicators, time‐temperature indicators, and gas indicators (Fuertes et al., 2016). According to our investigations, pH indicators are the only indicators coated within electrospun nanofibers, which have been prepared to be applied in food packaging. Some recent studies concerning the application of electrospun nanofibers containing indicators are given in Table 2.

**TABLE 2:** Application of electrospinning in intelligent packaging containing pH‐based indicators

Indicator	Concentration of indicator	Polymer	Solvent	Processing conditions	Average diameter (nm)	References
Red cabbage	15% w/v	Chitosan/ polyvinyl alcohol	Distilled water	15 kV, 6 ml/hr, 15 cm	‐	Jung et al. (2019)
Red cabbage	30% w/v	Zein	Ethanol (75% v/v)	16 kV, 1 ml/hr, 16 cm	444–510	Prietto et al. (2018)
Phycocyanin (microalgae)	9% w/v 3% w/v	Poly lactic acid poly ethylene oxide	Chloroform: Dimethylformamide (9:1 v/v)	15 kV, 0.6 ml/hr,14 cm	921–1318	Moreira, Terra, Costa, and de Morais (2018)
Red cabbage	10% w/v	Polyvinyl acohol	–	10,15, 20, 25 kV, 0.5, 0.7, 1, 1.5, 2 ml/hr, 10, 15, 20 cm	255–749	Maftoonazad and Ramaswamy (2019)
Açaí fruit	11% w/v 6% w/v	Polycaprolactone polyethylene oxide	Chloroform/ methanol (3:1 v/v)	20 kV, 0.6 ml/hr, 12.5 cm	1635	da Silva, da Silveira Mastrantonio, Costa, and de Morais (2019)

#### 4.1.3 pH indicator

Typically, visual pH indicators, representing the freshness of food products are constituted of two important parts, solid support and a dye which is sensitive to pH alterations (Ghorbani, Kaffashi, Shokrollahi, Seyedjafari, & Ardeshirylajimi, 2015) Packaging containing a pH indicator is one of the novelties of this branch, which is gradually developing. These dyes usually originate from various sources of vegetables and fruits. As soon as a particular food starts its spoiling process, a pH change befalls. This change is one of the most important indicators of food quality. At the beginning of the deterioration process, due to the pH variations, the color of indicator changes in the packaging (Medina‐Jaramillo, Ochoa‐Yepes, Bernal, & Famá, 2017). In this case, a package that specifies the pH of food before buying or consumption is a great means, ensuring the safety and quality of products to the consumer.

Given the animal or plant nature of foodstuff, there is a little difference in the function of indicators. In the cases, the edible material is a vegetable or a fruit, direct changes of pH on the surface of materials leads to the variation in the color of indicator. For instance, Maftoonazad and Ramaswamy (2019) synthesized a sensitive and rapid detection system of pH changes in Rutab (fresh date fruits) throughout the storage using electrospun polyvinyl alcohol nanofibers incorporated with red cabbage extract (RCE) as a pH indicator. The process parameters were as follow: applied voltage: 10, 15, 20, and 25 kV, feed rate: 0.5, 0.7, 1, 1.5, and 2 ml/hr, and collector distance: 10, 15, 20 cm. The average diameter for PVA fibers was 255 ± 53.4 nm, whereas the diameters of PVA fibers having 10, 20, and 30% of RCE were 655, 677, and 749 nm, respectively. The optimum conditions found for producing fine nanofibers were 15 kV voltage, 1 ml/hr flow rate, and 15 cm distance between needle and collector. After 72 hr, all aforementioned percentages of RCE incorporated in optimized nanofibers possessed violet color, indicating the spoilage of date due to the pH decrease to about 6. After 96 hr, their color changed to purple and pH decreased sharply (about pH of 5.3), resulting in the complete fruit spoilage at 25°C. These findings showed the possibility of designing a system, enabling a real‐time monitoring of pH changes in Rutab.

By the time, there is a need to package a protein base food, the increase in the total volatile base nitrogen (TVB‐N) indirectly causes the color change of indicator through increasing the pH. Total volatile base nitrogen is a collection of biogenic amines, including ammonia, dimethylamine (DMA), and trimethylamine (TMA), formed in nonfermented foodstuff in the course of storage. This index is widely used as a freshness criterion of different protein food products, especially seafood quality (Shukla, Kandeepan, Vishnuraj, & Soni, 2016, Ahmad, Heng, Salam, & Hanifah, 2018, Prabhakar, Vatsa, Srivastav, & Pathak, 2020). Microbial growth is a main cause of TVB‐N production. In fact, TVB‐N rise mostly is caused by decarboxylation of proteins by bacteria. The microbiological degradation of Trimethylamine N‐oxide (TMAO) to TMA and ammonia is the primary step in this process. All these volatile base nitrogen engender alkaline conditions.

Guo et al. (2020) developed double layer nanofiber mats composed of a colorimetric fiber layer (pullulan‐purple sweet potato extract [PL‐PSPE]) and an antibacterial layer (zein‐glycerol‐carvacrol [ZN‐GL‐CA]) using the electrospinning technique to detect the pH changes of pork. PSPE is a halochromic dye that changes color over the pH range of 2–3 to 7.2. Optimum fibrous mats were made at voltage of 9 kV, feed rate of 0.7 ml/hr, and distance from tip‐to‐collector of 12 cm. The fabricated nanofibers were able to determine changes of pH as a result of variations in color of sweet potato extract. The mat color changed from purple and blue to green then, the color reversed. The change in ΔE values of double layer mats was 7.56, providing faster and more sensitive detection.

Aghaei, Ghorani, Emadzadeh, Kadkhodaee, and Tucker (2020) produced zein electrospun nanofibers covering alizarin as a halochromic indicator based on TVB‐N for controlling the freshness of rainbow trout fillets throughout the storage time. They regulated the electrospinning process to 20, 25, and 30 kV voltage, 1 ml/hr flow rate, and 12 cm distance between the needle tip and collector. The average diameter of formed fibers was 79–619 nm. The fibers exhibited a yellow color at the beginning, when the TVB‐N was nearly 17 mg/100 g food sample, then light purple color on day 6 (TVB‐N of approximately 23 mg/100 g food sample), and finally magenta color on day 12 (TVB‐N of approximately 33 mg/100 g food sample). These color changes toward getting darker indicated that the fabricated system could successfully monitor the freshness of fillets by evaluation of TVB‐N amount.

In a similar survey, Aghaei, Emadzadeh, Ghorani, and Kadkhodaee (2018) developed a halochromic system composed of cellulose acetate nanofibres synthesized by electrospinning method (voltage: 25 kV, flow rate: 0.5 ml/hr, tip–collector distance: 15 cm) containing alizarin. They investigated the fish spoilage at the refrigerator temperature for 12 days. The color changes were strongly influenced by increasing the amount of TVB‐N of product. They found that no color change was observed in the first 48 hr, when the TVB‐N slightly increased from 11 to 13 mg/100 g sample, but after this, the color changed to very light brick at the TVB‐N level of 18 mg/100 g sample, and at the end of the period it changed to violet (TVB‐N of 22 mg/100 g sample).

## 5 CONCLUSION

Electrospinning technique has been developed significantly during recent few years. This method is known as an efficient technique to produce nanofibers with high surface to volume ratio, absorbance capacity, porosity, and small pore size. These characters make electrospun nanofibers possible to be accompanied with different food spoilage indicators in an intelligent packaging. All these advantages lead to fast response, easy‐to‐visualize, and real‐time freshness monitoring of foodstuff. Given the novelty of intelligent packaging possessing electrospun nanofibers, the most of such packaging produced in recent years are pH indicators. Therefore, the development of these packages to monitor compounds such as ethanol is predictable. According to our knowledge, two important issues are missing throughout the studies, which may be addressed in future researches. First, there was not any report to compare the effectiveness of indicators in packages prepared with the help of electrospinning technique and other usual packages. Second, almost the majority of such packaging have been developed together with indicators derived from fruits and vegetables and none of them did not benefit from the help of a sensor, which could exactly and quantitatively monitor the characteristics of foodstuff may be due to the economic issues.

## CONFLICT OF INTEREST

The authors declare that there is no conflict of interest.

## ETHICAL APPROVAL

The study did not involve any human or animal testing.
